# Hakai overexpression effectively induces tumour progression and metastasis *in vivo*

**DOI:** 10.1038/s41598-018-21808-w

**Published:** 2018-02-22

**Authors:** Raquel Castosa, Olaia Martinez-Iglesias, Daniel Roca-Lema, Alba Casas-Pais, Andrea Díaz-Díaz, Pilar Iglesias, Isabel Santamarina, Begoña Graña, Lourdes Calvo, Manuel Valladares-Ayerbes, Ángel Concha, Angélica Figueroa

**Affiliations:** 1 Epithelial Plasticity and Metastasis Group, Instituto de Investigación Biomédica de A Coruña (INIBIC), Complexo Hospitalario Universitario de A Coruña (CHUAC), Universidade da Coruña (UDC), Sergas, Spain; 2 Pathology Department, INIBIC, CHUAC, Sergas, UDC Spain; 3 Clinical and Translational Oncology Group, INIBIC, CHUAC, Sergas, UDC Spain; 4 Department of Medical Oncology, Hospital Universitario Reina Sofía, Córdoba, Spain

## Abstract

At early stages of carcinoma progression, epithelial cells undergo a program named epithelial-to-mesenchymal transition characterized by the loss of the major component of the adherens junctions, E-cadherin, which in consequence causes the disruption of cell-cell contacts. Hakai is an E3 ubiquitin-ligase that binds to E-cadherin in a phosphorylated-dependent manner and induces its degradation; thus modulating cell adhesions. Here, we show that Hakai expression is gradually increased in adenoma and in different TNM stages (I-IV) from colon adenocarcinomas compared to human colon healthy tissues. Moreover, we confirm that Hakai overexpression in epithelial cells drives transformation in cells, a mesenchymal and invasive phenotype, accompanied by the downregulation of E-cadherin and the upregulation of N-cadherin, and an increased proliferation and an oncogenic potential. More importantly, for the first time, we have studied the role of Hakai during cancer progression *in vivo*. We show that Hakai-transformed MDCK cells dramatically induce tumour growth and local invasion in nude mice and tumour cells exhibit a mesenchymal phenotype. Furthermore, we have detected the presence of micrometastasis in the lung mice, further confirming Hakai role during tumour metastasis *in vivo*. These results lead to the consideration of Hakai as a potential new therapeutic target to block tumour development and metastasis.

## Introduction

Carcinoma is the most common type of cancer and arises from the transformation of epithelial cells. Around 90% of cancer related deaths are consequence of metastasis. At early stages of tumour progression and carcinoma metastasis, epithelial tumour cells activate a crucial program named epithelial–to–mesenchymal transition (EMT), which is frequently observed in human carcinoma. EMT is a highly controlled program firstly reported during embryogenesis (EMT type I), and also described in wound healing and tissue repair (EMT type II). Apart from these physiological EMT, this program can also occur during pathological conditions such as organ fibrosis and tumour progression (EMT type III). Cancer-EMT is characterized by the disruption of cell–cell contacts, cell–substratum adhesions and apical–basal polarity, accompanied by the reorganization of the cytoskeleton. All these changes cause the loss of epithelial phenotype and the acquisition of a mesenchymal phenotype, which includes a gain of migratory and invasive capabilities, important for the dissemination of cancer cells^1–3^. One of the best-characterized hallmarks of the EMT is the loss of E-cadherin in epithelial cells^4–6^. E-cadherin is the prototype member of classical cadherins at adherens junctions in epithelial cells, and its loss is associated to the progression from adenoma to carcinoma therefore, from benign tumour to malignant tumour^6,7^. In addition to the loss of the epithelial E-cadherin protein, the mesenchymal marker N-cadherin is upregulated during EMT, and this switch between cadherins is also considered as a hallmark of cancer-related EMT program^8^.

It has been extensively studied the mechanism whereby E-cadherin is downregulated during EMT in cancer^9,10^. Hakai is the first post-translational regulator described for the E-cadherin stability^11^. Hakai, a new class of the three families of RING-finger type E3 ubiquitin-ligases, contains a novel domain called HYB (Hakai pTyr-binding) whereby interacts with the tyrosine-phosphorylated E-cadherin by Src, inducing its ubiquitination and degradation, which in turn causes the alteration of cell–cell contacts^11–13^. The ubiquitination system frequently signals for protein degradation into lysosome or proteasome pathway system. It is generally believed that cytosolic and nuclear proteins are mainly degraded via proteasome, while membrane proteins are routed for destruction into lysosome^14^. Indeed, lysosomal targeting of E-cadherin is the described mechanism for the downregulation of cell–cell adhesion during EMT^15^. In addition to Hakai action on cell–cell contacts, it has been described its involvement in the reduction of the cell–substratum adhesions and the increase of epithelial cell invasion *in vitro*^16^. Moreover, we have previously reported that Hakai expression is increased in human colon and gastric adenocarcinoma compared to its adjacent healthy epithelial tissue, further supporting Hakai role during tumour progression^17–19^. Hakai is also involved in the regulation of cell proliferation in an E-cadherin-independent manner, suggesting that ubiquitinated novel substrates by Hakai wait to be elucidated^17^. Indeed, Cortactin and DOK1 were recently identified as a novel Hakai-interacting and ubiquitinated proteins in Src-phosphorylation-dependent manner. However, the physiological relevance of these interactions is not defined yet^12^.

Despite Hakai ubiquitin-dependent functions, it has been proposed that Hakai may be involved in several cellular processes in an ubiquitin-independent manner^17,20–23^. According to this, in a proteomics analysis of *Arabidopsis thaliana*, Hakai was identified as an interacting protein of several post-transcriptional regulators, including *N*6-methyladenosine (m^6^A) writer complex members^24^. m^6^A is an important mRNA modification found in eukaryotes which is involved in processes such as splicing, mRNA stability, mRNA export and others. In this model, Hakai is functionally necessary for mRNA methylation, suggesting a possible similar role in mammals^25^. On the other hand, Hakai interacts with the RNA-binding protein PSF (*Polypyrimidine tract binding protein associated Splicing Factor*). Indeed, Hakai overexpression increase the binding of PSF to mRNA transcripts encoding specific cancer-related proteins^17,20^. Up to date, most of the publications related to the role of Hakai on E-cadherin expression were reported in an *in vitro* model system. Apart from the described role of Hakai in *A. thaliana*, other *in vivo* study was performed in *Drosophila melanogaster* where Hakai function was described to be most crucial at early stages of embryogenesis^26^. In our study, we have extended our previous results to examine Hakai function in cancer progression by using human tissue samples from patients at different stages of colon cancer progression and the Hakai functional role *in vivo* by using a mice xenograft tumour model. By using Madin-Darby Canine Kidney (MDCK) cells, an established model system to study cell-cell adhesions, we have reported that Hakai overexpression induces tumour progression and micrometastasis *in vivo*.

## Results

### Hakai expression levels in different colon cancer TNM stages

**Figure 1:**
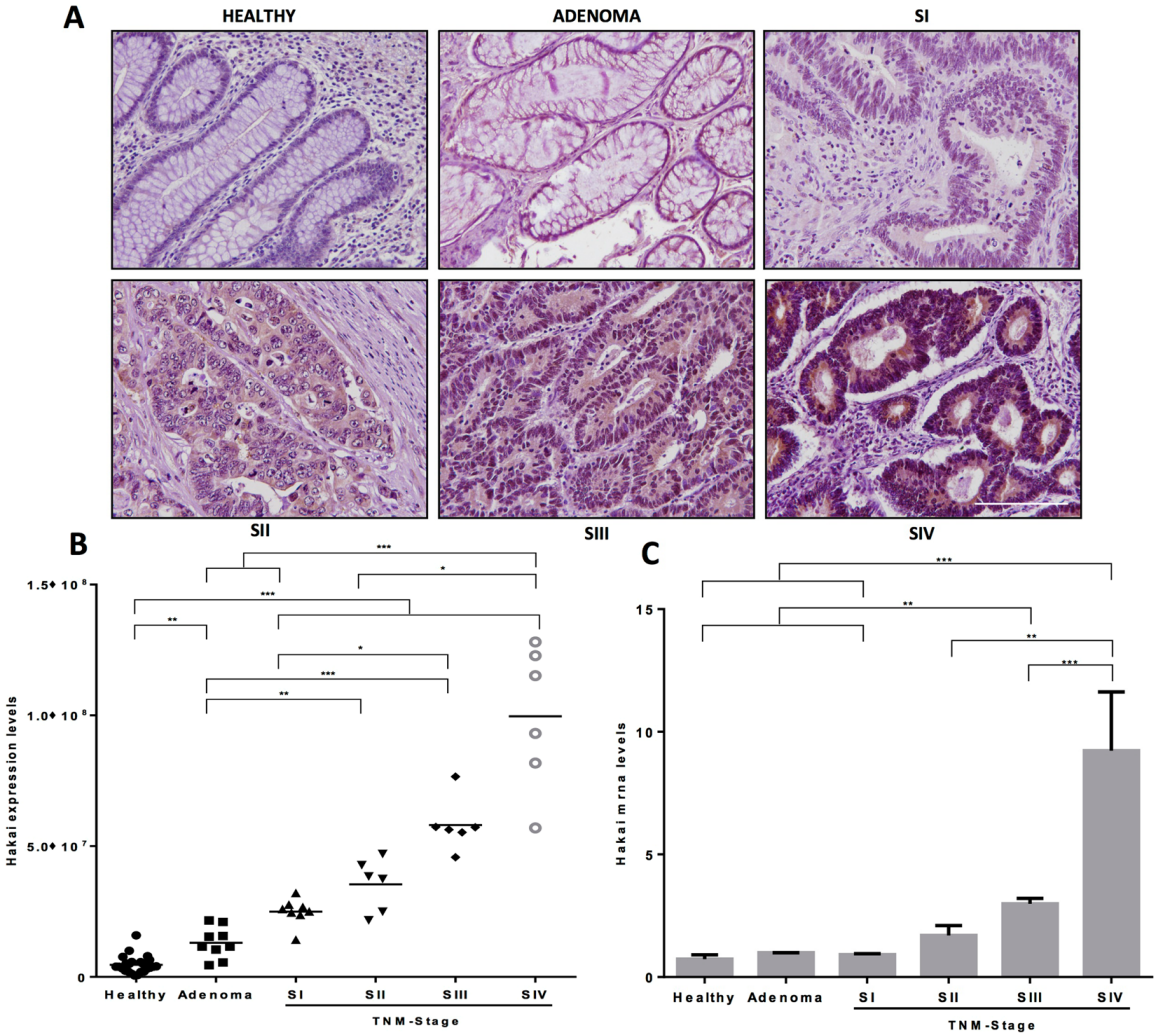
Hakai expression levels in human samples from colorectal cancer patients. (**A**) Representative immunoreactivity of Hakai in normal colonic mucosa, adenoma, and colorectal cancer (TNM stages I-IV). Images were obtained with a 20x objective. Scale bar 125 μm. **(B)** Statistical quantification of Hakai staining intensity in epithelial cancer cells at different colon cancer stages and in adenoma and normal colon tissues (normal colonic mucosa, *n* = 26; adenoma, *n* = 10; colorectal cancer, *n* = 20 of all stages). Five photographs of each tissue were quantified. Data are represented as scatter plot. Values are means ± SEM of staining intensity signal scoring per area. Calibration and quantification of the images were performed with ImageJ software. Kruskal-Wallis with Tukey correction test analyses show statistical differences in colorectal cancer (TNM, SI-IV) respect to paired healthy samples (*p < 0.05; **p < 0,01; ***p < 0,001). **(C)** Hakai mRNA expression levels normalized to control RPL13A mRNA were measured in normal colonic mucosa, adenoma, and colorectal cancer (normal colonic mucosa, *n* = 12; adenoma, *n* = 3; colorectal cancer, *n* = 12 of all stages).

By using human colon carcinoma samples, we have previously reported that Hakai expression levels were higher in colon cancer tissues than in adjacent normal epithelial tissues^19^. Here, we extend our previous analysis comparing pairs of human colon healthy tissues to adenoma and to different TNM stages (I-IV) from colon adenocarcinomas, as shown in a representative image (Fig. 1A). The quantification of the signal intensity of Hakai immunohistochemistry staining is shown in Fig. 1B. Staining intensities of Hakai in cancer cells were gradually increased in all stages I-IV compared to healthy normal epithelium. Moreover, the mesenchymal N-cadherin marker is upregulated in advance colon carcinoma progression compared to healthy colon tissues and the E-cadherin loss is associated to the progression from adenoma to carcinoma. However, we did not detect significant differences in protein expression in the other reported substrate for Hakai, Cortactin, during colon carcinoma development (Supplementary Fig. 1). Furthermore, we have also analysed Hakai mRNA levels showing a significant increase in TNM stage III and IV compared to healthy tissues, indicating that the transcriptional regulation of mRNA Hakai may be a late event during tumour progression (Fig. 1C). Taken together our results suggest the potential use of Hakai as novel biomarker for colon cancer progression.

### Hakai promotes tumour formation and proliferation *in vivo*

**Figure 2:**
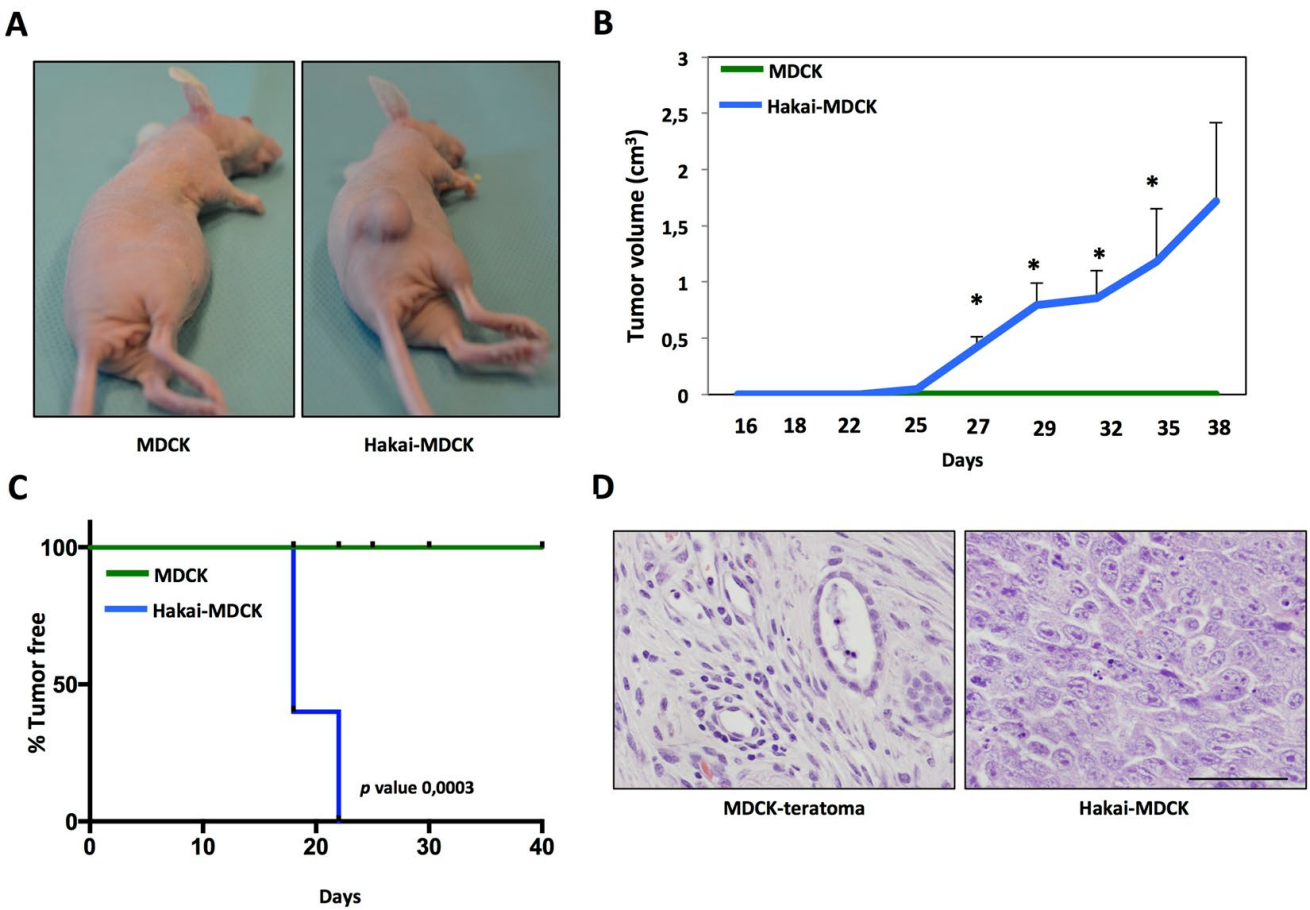
Hakai induces tumour formation *in vivo*. **(A)** Representative photographs after 38 days of subcutaneous injection of 5 × 10^6^ MDCK or Hakai-MDCK into nude mice cells (MDCK, *n* = 4; Hakai-MDCK, *n* = 5). **(B)** Tumour growth in nude mice injected with MDCK or Hakai-MDCK. Tumours were measured twice a week, as described in Materials and Methods and results are represented as mean ± SEM (*P < 0,05). **(C)** Tumour-free survival of MDCK or Hakai-MDCK cells in nude mice. *p* value was calculated with Breslow test and is indicated in the figure. **(D)** Representative H&E staining of teratoma and tumours originated by MDCK or Hakai-MDCK cells, respectively. Pictures were taken with a 40x objective, scale bar 500 μm.

Given that Hakai expression in human colon adenocarcinoma is highly upregulated compared to normal tissues suggesting its contribution to tumour progression, we decided to further study the possible role of Hakai during tumour progression *in vivo*. For this purpose, we used an established system, a normal epithelial Madin-Darby Canine Kidney (MDCK) cell line^27,28^. As previously reported, we confirmed that Hakai overexpression in MDCK cells (Hakai-MDCK clone 4 and clone 11) transformed the normal epithelial phenotype of MDCK into a mesenchymal morphology^17^ (Supplementary Fig. 2A), accompanied by the decrease of protein expression of Hakai substrates, E-cadherin and Cortactin, and a robust increase of the mesenchymal marker N-cadherin (Supplementary Figs 2B and 3). As mentioned, this cadherin switch (between E- and N-cadherins), observed when Hakai is overexpressed in MDCK cells, is a classical event seen during cancer-related EMT program. Moreover, we confirmed that Hakai-MDCK cells acquire the ability to invade and exhibit oncogenic potential (Supplementary Fig. 2C, D). On the other hand, important efforts were made to obtain stable transfection to knocking-down Hakai in several epithelial cell lines, however, we failed to do so suggesting that Hakai protein may be crucial for cell survival. MDCK and Hakai-MDCK cells were injected subcutaneously into the flank of nude mice to get further insights into the possible role of Hakai during tumour progression *in vivo*. Hakai-MDCK cells formed primary tumours in all injection sites whereas parental MDCK cells were unable to do so, as shown in Fig. 2A. Tumour growth induced by Hakai-MDCK cells was measured showing an increased tumour volume 38 days post-injection. Palpable tumours were detected in the mice injecting 5 × 10^6^ Hakai-MDCK cells on day 18 post-injection (Fig. 2B). All mice injected with MDCK cells were tumour-free, on the contrary, local tumour in Hakai-MDCK inoculated mice appeared after 18 days and all mice showed tumours after 22 days post injection. After 38 days all mice were euthanized (Fig. 2C). Histological analysis of the tumour xenografts further confirmed the biological effect of Hakai overexpression. Interestingly, while injecting 5 × 10^6^ MDCK cells, we observed a teratoma formation (Fig. 2D), however, these findings were not observed by injecting 1 × 10^6^ MDCK cells (data not shown). Our results are in concordance with previous reported results on which adjacent MDCK cells maintain the ability to regenerate kidney tubule-like structures *in vivo* by using athymic nude mice, keeping joined junctions and retained regional differentiation^29^. In contrast, tumours induced by Hakai-MDCK cells showed a significant change to undifferentiated and spindle-shape carcinoma cells. Cell morphology of Hakai-MDCK xenografts was dramatically changed showing an increase in nucleus size with an irregular size and shape, and prominent nucleoli. On the contrary, a reduction of the cytoplasm size is seen. All these morphological changes are characteristic of cancerous cells (Fig. 2D).

**Figure 3:**
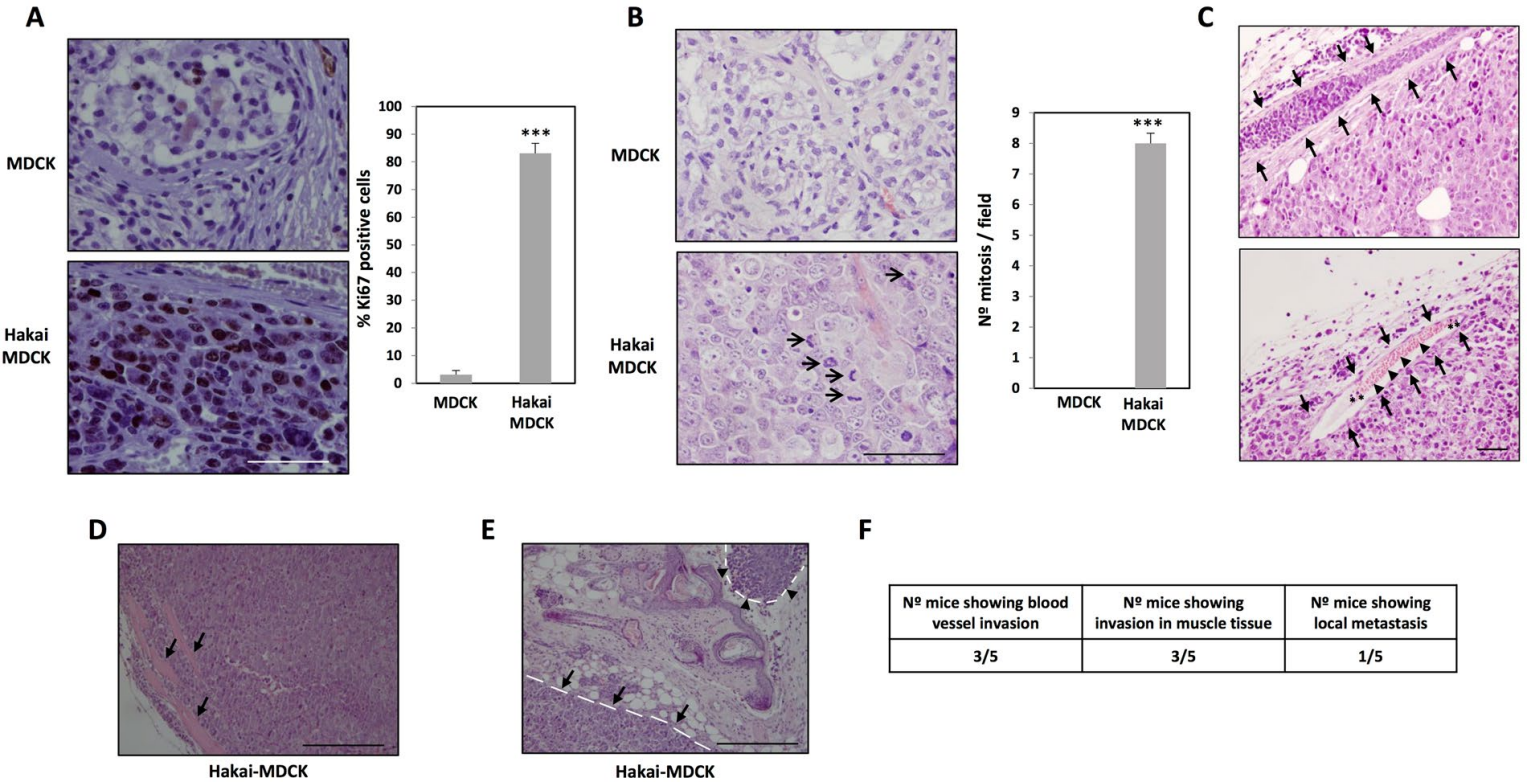
Hakai action on tumour proliferation and invasion in a mouse xengraft model. **(A)** Immunohistochemistry for ki67 expression in teratoma and tumours originated by MDCK or Hakai-MDCK cells, respectively (MDCK, *n* = 2; Hakai-MDCK, *n* = 5, five pictures per each were taken). Representative images were taken with the 40x objective (*left panel*) and the quantification of the percentage of positive cells is shown (*right panel*). Scale bar, 500 µm. **(B)** Mitotic cells in MDCK and Hakai-MDCK xenografts (MDCK, *n* = 2; Hakai-MDCK, *n* = 5, five pictures per each were taken). Mitotic cells in representative images of H&E staining taken with a 40x objective (*left panel*, *arrows*) and the corresponding mitosis quantification in xenografts (*right panel*). Scale bar, 500 µm. **(C)** H&E staining in the upper panel shows an infiltrated blood vessel with tumour cells originated by Hakai-MDCK injection, and in the bottom panel a blood vessel containing leukocytes (asterisks) and erythrocytes (arrowheads) is shown. Blood vessels are indicated with arrows. Photograph was taken with a 20x objective. Scale bar, 100 μm. **(D)** H&E staining showing muscle infiltration by Hakai-MDCK cells. Picture was taken with a 10x objective. Muscle is indicated with arrows. Scale bar, 500 μm **(E)** H&E staining showing primary tumour (arrows) and a local metastasis (arrowheads) originated by Hakai-MDCK cells are indicated with a discontinuous line. Connective tissue is found between the primary tumour and the local metastasis. Picture was taken with a 10x objective. Scale bars, 500 µm. Quantifications are shown as mean ± SEM (***P < 0,001). **(F)** Table including the number of animals containing infiltrated tumour cells in blood vessels (**C**), in muscle tissue (D) or a local metastasis (**E**). H&E stained sections were examined under microscopy and the number of animals with tumour cells into blood vessels, muscle tissue or with local metastasis were counted.

Growth potential quantification of these tumours was characterized by studying Ki67 immunohistochemistry and mitotic index. Tumours originated by Hakai-MDCK cells were highly proliferative with more than 80% of positive cells for Ki67 labelling (Fig. 3A). These results were confirmed with the quantification of mitotic index, a parameter commonly used in clinical diagnosis, showing an extraordinary high number of cells in mitosis detected in Hakai-MDCK tumour sections stained with H&E (Fig. 3B, arrows). In conclusion, a marked increase of both proliferation markers in Hakai-MDCK tumours was observed supporting that Hakai-MDCK cells arise tumours with an extremely high proliferative rate.

### Hakai induces local invasion and tumour cells exhibit a mesenchymal phenotype

**Figure 4:**
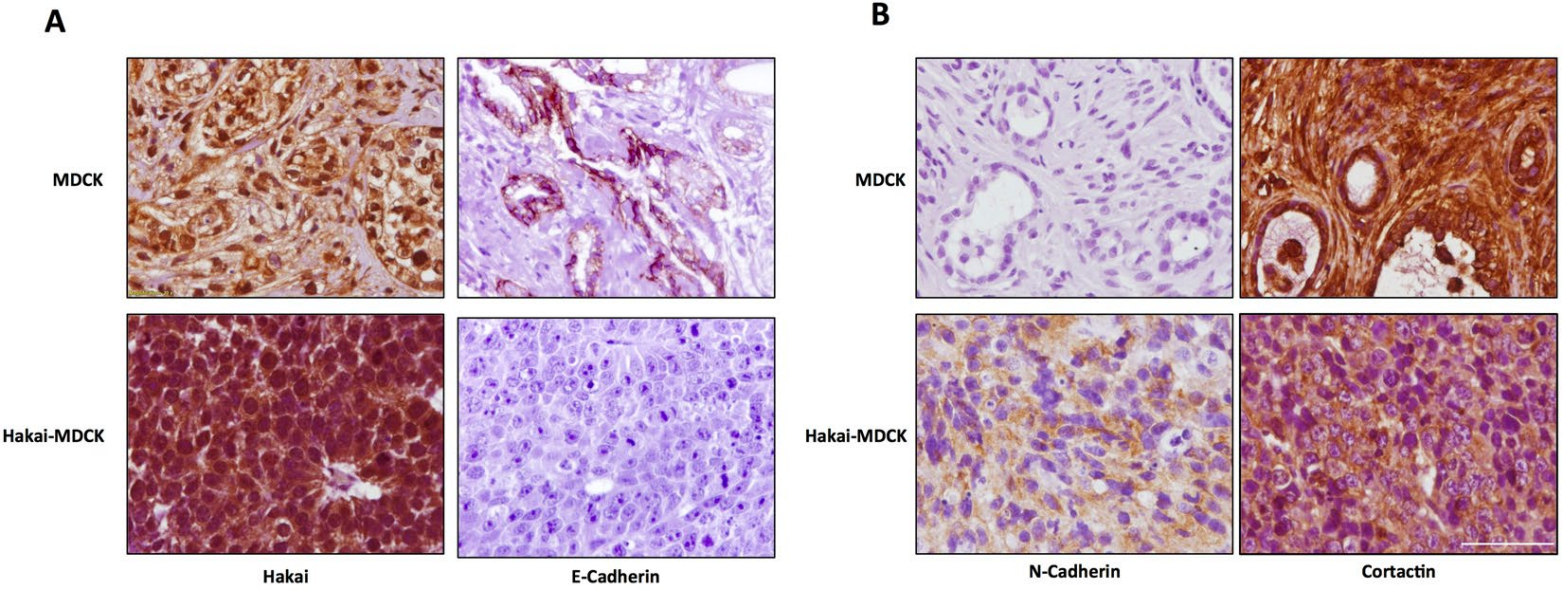
Hakai overexpression induces tumours with mesenchymal phenotype in xenografts mouse model. **(A)** Immunohistochemical staining for E-cadherin and Hakai in tumours originated by MDCK and Hakai-MDCK cells (MDCK, *n* = 2; Hakai-MDCK, *n* = 5). Representative images were taken with a 40x objective. Scale bar, 125μm. **(B)** Representative immunoreactivity of N-cadherin and Cortactin in tumours originated by MDCK or Hakai-MDCK cells (MDCK, *n* = 2; Hakai-MDCK, *n* = 5). Representative images were taken with a 40x objective. Scale bar, 125 μm.

In order to further evaluate the *in vivo* role of Hakai during invasion and metastasis, we first performed H&E staining of the tumour sections. After 38 days, blood vessels were infiltrated with tumour Hakai-MDCK cells (Fig. 3C, upper panel), erythrocytes or leukocytes (Fig. 3C, lower panel), although no specific immunoreactivity was detected using anti-HA antibody to specifically detect of Hakai-MDCK (Supplementary Fig. 4). It was determined that 60% of the mice showed infiltrating Hakai-MDCK cells in blood vessels (Fig. 3C), and invading the adjacent muscle (Fig. 3D) further supporting the implication of Hakai on invasion *in vivo*. Furthermore, 20% of the mice showed an extent of primary tumour invasion (Fig. 3E), a fact of particular relevance which suggests the extremely aggressive behaviour of the Hakai-MDCK cells, as mice with locally invasive tumours are more likely to develop metastases and also tend to have a worse prognosis. Then, we also studied the possible Hakai influence on E-cadherin-mediated cell-cell contacts. First, we confirmed that Hakai-MDCK cells in xenograft mouse model continued to express higher Hakai levels compared to the MDCK injected cells (Fig. 4A, *left panel* and Supplementary Fig. 5A, *left panel*). We also analysed E-cadherin expression as its loss is a prerequisite and hallmark of EMT, and critical for the invasive and malignant phenotype^30,31^. The expression of the epithelial marker E-cadherin was found to be completely disappeared in Hakai-MDCK injected cells *in vivo*, whereas remaining E-cadherin at cell-cell contacts was detected in the teratoma formation by MDCK-injected cells (Fig. 4A, *right pane* and Supplementary Fig. 5A, *right panel*). Moreover, Hakai-MDCK tumours were robustly positive for N-cadherin mesenchymal marker whereas MDCK teratomas were negative (Fig. 4B, *left panel* and Supplementary Fig. 5B, *left panel*). This cadherin switch was described as a a hallmark of cancer-related EMT program^8^. Finally, we also extended our study by analysing the expression of another *in vitro* described substrate for Hakai, Cortactin. As expected, Cortactin expression was reduced in Hakai-MDCK xenograft tumours compared to MDCK-injected cells, supporting *in vivo* the previous *in vitro* reported action of Hakai in the ubiquitination and degradation of Cortactin (Fig. 4B, *right panel* and Supplementary Fig. 5, *right panel*)^12^. Interestingly, Hakai-MDCK xenograft tumours show Cortactin expression only in the cytoplasm, whereas in MDCK teratoma is also highly enriched in the nucleus. Taken all together these results indicate that Hakai-MDCK exhibits a mesenchymal phenotype and induce local invasion *in vivo*.

### Hakai overexpression produces lung micrometastasis *in vivo*

**Figure 5:**
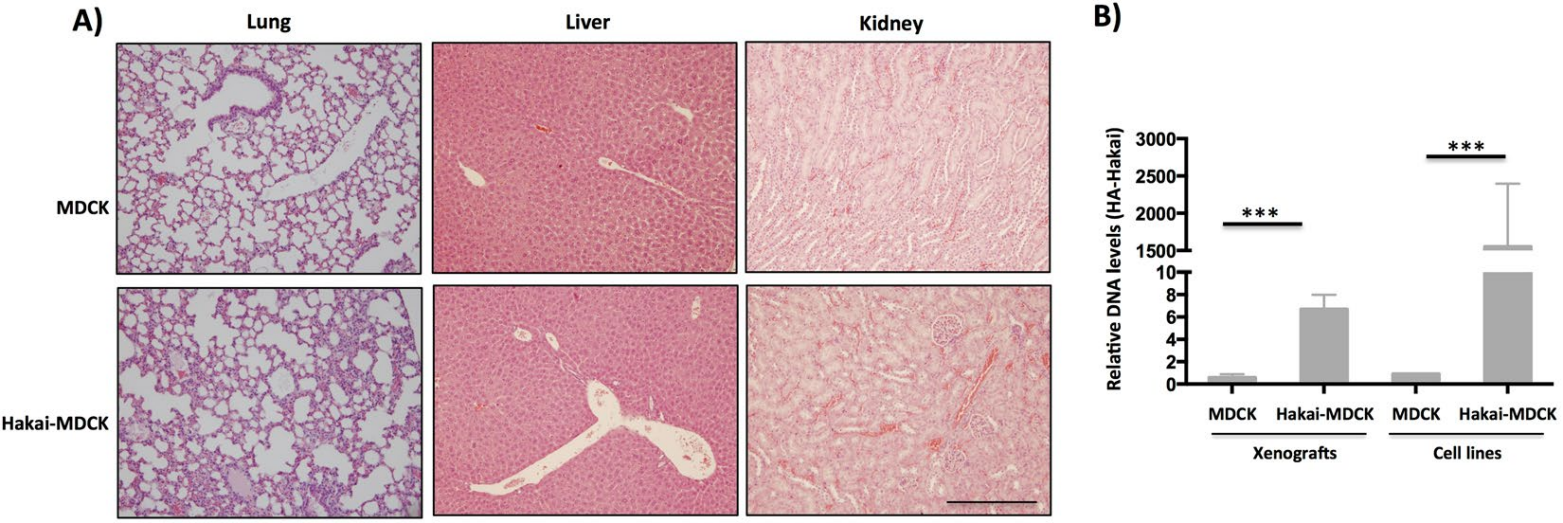
Hakai induces lung micrometastasis in nude mice. **(A)** Analysis of macrometastasis presence in lung, liver and kidney. MDCK or Hakai-MDCK cells were inoculated into the flank of nude mice and after 38 days the indicated organs were excised, fixed and stained with H&E in order to detect macrometastasis. Representative pictures were taken with a 10x objective. Scale bar, 500 μm. **(B)** The presence of tumour cells into the mice lung was assessed by quantitative PCR analysis of HA-Hakai sequences as indicated in Materials and Methods. Tumour xenografts from MDCK and Hakai-MDCK injected cells (*n* = 4 and *n* = 5 respectively) were measured and MDCK and HA-MDCK cell line were used as negative and positive controls, respectively (***P < 0,001).

Finally, to determine whether Hakai may promote cancer metastasis, lung, kidney and liver tissues were analysed by H&E staining. Metastasis to distant sites was not observed neither with Hakai-MDCK nor MDCK cells (Fig. 5A). This result was probably due to short timing analysis for the *in vivo* study, as 38 days after injection of cells may not be enough to allow cells to settle down and form macrometastasis. Moreover, no specific signal was detected in lung and liver tissues by using HA antibody (Supplementary Fig. 6). Therefore, we decided to further determine the possible existence of micrometastasis by analysing the presence of DNA of Hakai-MDCK in lung. For this purpose, HA-tagged Hakai present in Hakai-MDCK cells was measured by using two different specific primers: one designed for HA epitope and the second primer for Hakai. After 38 days post-injection, micrometastases were detected in the lung of the Hakai-MDCK xenograft mouse model while no detection was found in MDCK cells xenografts. MDCK and Hakai-MDCK cell lines were used as negative and positive controls respectively (Fig. 5B), further confirming that Hakai induces lung micrometastasis. Taken together, our results underscore an important role of Hakai during tumour progression and metastasis *in vivo*.

## Discussion

In this study, we describe for the first time the role of Hakai *in vivo*. Our findings indicate that Hakai protein expression gradually increases during human colon cancer progression. Interestingly, in benign colon adenoma samples, protein expression is statistical enhanced compared to normal tissues. These data suggest that Hakai may not only be involved in the malignant progression of human colon cancer but also at early stages of tumorogenesis by acting on cell proliferation^17^. When analysing Hakai mRNA levels, it is shown a statistically significant increase in TNM-stages III and IV compared to normal and adenoma human colon tissues, further suggesting that the regulation of Hakai at transcriptional level may be a late event during tumour progression. Therefore, our data highlight the potential use of Hakai as a biomarker during colon cancer progression. However, further investigations are required to elucidate whether Hakai expression could be correlated with patient survival and/or associated with poor outcome or other clinic-pathologic characteristics of colorectal cancer patients.

In keeping with previous reports, during EMT the loss of the epithelial E-cadherin protein is accompanied by an upregulation of the mesenchymal marker N-cadherin. Loss of E-cadherin–mediated cell adhesion coincides with the transition from well differentiated adenoma to invasive carcinoma in a transgenic mouse model^7^. We also confirm the downregulation of E-cadherin and upregulation of N-cadherin during colon cancer progression. Our results also show that no loss of E-cadherin was observed in human colon adenoma compared to normal healthy adjacent tissues, as previously reported^32^. Therefore, other levels of regulation could be involved, as for example, the loss of E-cadherin may be regulated by hypermethylation on E-cadherin promoter at early event during colon cancer development^33^. As Hakai expression is gradually increased during tumour progression, our results underscore that Hakai could contribute to an invasive phenotype in colon adenocarcinoma by downregulating E-cadherin at early stages of tumour progression. Moreover, our group has previously published that knocking-down Hakai, by using a small interference RNA, blocked proliferation in MCF7 and HEK-293 cells, accompanied by the reduction of the expression of the important cell cycle regulator cyclin D1. Furthermore, by using a mutant of the RING-finger domain (Hakai-ΔRING), necessary for Hakai E3 ubiquitin-ligase activity, we showed that this domain was necessary for Hakai effect on proliferation. According to the demonstrated role of Hakai E3 ubiquitin-ligase activity on proliferation *in vitro*, it was studied Hakai expression in two different human tissues. Certainly, increased Hakai expression was detected in the germinal centres of lymph node, where lymphocytes are actively proliferating, and in the proliferative phase of the endometrium, while low expression was observed in the secretory phase^17^. Moreover, it has been also published that Wilm’s tumor 1-associating protein (WTAP) forms a complex with several proteins, including Hakai, which is required for cell cycle progression. Indeed, silencing Hakai expression in this model with a small interference RNA reduced proliferation with G2/M accumulation^24^. Moreover, by analysing the functional role of the Hakai homologue in *Drosophila melanogaster*, *Kaido et. al*. demonstrated that Hakai null mutants died during larval stages^26^.

Interestingly, it has been suggested that Hakai protein may be particularly relevant at early stages of EMT. *Janda et. al*. reported that the cooperation between Ras and TGFβ to activate EMT enhanced E-cadherin endocytosis and lysosomal degradation. They demonstrated that E-cadherin is downregulated at post-translational level at initial phases of EMT whereas the loss of E-cadherin via transcriptional repression is a late event during EMT^34^. On the other hand, in *Droshophila melanogaster*, it was demonstrated that Hakai function is most vital at early stages of embryogenesis and its contribution decreases at later stages^26^, and in *Arabidopsis thaliana*, Hakai is required for methylation of mRNA (m6A)^25^, which is described as an essential process for the earliest stages of pattern formation in plants^35–37^. All these publications further support that Hakai may act on early stages of EMT during carcinoma progression by its action as a post-translational regulator of E-cadherin. However, novel research lines are needed to determine whether Hakai may act on other novel unidentified substrates or as a post-transcriptional regulator by its influence on mRNA stability, m6A process or others, given that in different model systems Hakai is mainly localized in the nucleus^17,25,26^. Therefore, future investigations regarding to the molecular mechanism by which Hakai influences carcinoma progression will contribute to open a novel direction for therapeutic intervention against cancer.

Cortactin is a cytoskeleton protein and one of the major substrates for Src kinase. It is required for cell migration and it is present in cell-motility structures such as lamellipodia and invadopodia, opening a great interest in tumour invasion. Although cortactin is overexpressed in many types of cancers such as head and neck squamous carcinoma, oral squamous carcinoma, breast cancer or melanoma, we could not find any regulation in protein levels in human colon cancer tissues^38–41^. It has been reported that subcellular localization and activity is determinant for cortactin-mediated cell migration. Indeed, it was shown that cortactin is regulated by various post-translational modifications, such as acetylation. *Ito et. al*. demonstrated that acetylated form of cortactin is mainly localized in the nucleus and this acetylated-cortactin decreases cell migration by inhibiting its binding to Keap1 protein in the cytoplasm^42,43^. These interesting findings elucidate for the first time the opposite effect on cellular migration depending on the subcellular localization of cortactin. Therefore, it is plausible that Hakai may also influence the migratory capabilities that are important for the dissemination of cancer cells by its action on cortactin localization.

Given the important reported role of the E3 ubiquitin-ligase Hakai during carcinoma metastasis *in vitro* and *in vivo*, the development of novel inhibitors against the E3 ubiquitin-ligase Hakai appears to be an attractive strategy for therapeutic interventions. So far, the FDA has approved very few drugs targeting members of the ubiquitin pathway, which include the proteasome inhibitors bortezomib, carfilzomib and ixazomib. However these drugs are limited to specific hematopoietic malignancies, and clinical trials testing their use for solid tumours have been so far disappointing^44,45^. Indeed, a recent publication demonstrated that in epithelial cells undergoing EMT, the proteasome activity is decreased and the proteasome inhibitors induced EMT^46^. According to this, we have recently demonstrated a specific downregulation of several proteasome subunits in Hakai-MDCK epithelial cells compared to non-transformed MDCK cells^23^. Taken together and given that Hakai expression is enhanced in human tumour progression in colon adenocarcinoma compared to normal tissues (Fig. 1), our results reinforce that proteasome inhibitors may not be an effective treatment for epithelial tumours that follow EMT and we propose the E3 ubiquitin-ligase Hakai as a better therapeutic target against cancer in specific molecular subtypes of colorectal cancer already defined^47,48^.

## Materials and Methods

### Mice and human tissues samples

Animal experiments were performed in the Experimental Surgery Unit-Technological formation centre from INIBIC-CHUAC in compliance with the European Community Law (86/609/EEC) and the Spanish law (R.D. 53/2013), with approval of the Experimental Animal Ethics Committee from Xerencia de Xestión Integrada A Coruña (XXIAC). Colon cancer biopsies were obtained from the Pathological Anatomy department from the Complejo Hospitalario Universitario A Coruña (CHUAC), under informed consent from all patients signed and research investigation was approved by the Research Ethics Committee from A Coruña-Ferrol and performed following standard ethical procedures of the Spanish regulation (Ley Orgánica de Investigación Biomédica, 14 July 2007). Paraffin samples were provided by CHUAC Biobank integrated in the Spanish Hospital Platform Biobanks Network.

### Antibodies and materials

The rabbit polyclonal anti-Hakai antibody (Hakai-2498) was provided by Dr. Fujita. For immunochemistry Anti-E-cadherin antibody (24E10), from Cell Signaling, was used. Anti-Cortactin antibody (05-180) was from Millipore. Anti-N-cadherin (ab18203) was from Abcam. Anti-Ki67 monoclonal antibody (clone MIB-1, code M7240) was from DAKO. For nude mice immunohistochemistry, antibodies were used at dilution 1/400 for E-cadherin, 1/250 for Hakai, 1/150 for Ki67, 1/100 for N-Cadherin, 1/50 for Cortactin. For human immunohistochemistry Hakai dilution was 1/700.

### Cell lines

MDCK cells were cultured in Dulbecco’s Modified Eagle Medium (DMEM) containing 1% penicillin/streptomycin, 1% glutaMAX and 10% of heat-inactivated fetal bovine serum (FBS). MDCK stably expressing Hakai cells (Hakai-MDCK) were provided by Dr. Fujita^17^ and were cultured in presence of the selection antibiotic G418 (800 μg/ml). In the present work, Hakai-MDCK (clone 4) was used, however, all selected clones represented comparable phenotypes and results^17^. Cells were grown at 37 °C in a humidified incubator with 5% CO_2_. Cells were also tested regularly for mycoplasma contamination and all cells used were negative for mycoplasma test.

### Real-time quantitative PCR (qRT-PCR)

Ten sections of paraffin-embedded (FPPE) human colon cancer tissues (4 µm) were cut and deparaffinized in an eppendorf using xylene. Total RNA was extracted using the RNeasy FFPE Kit (Qiagen). Three adenomas, three colorectal cancer of every TNM stage and 12 healthy tissues were analysed. mRNA levels were analysed in technical triplicates by quantitative RT-PCR, following specifications of reverse retrotranscriptase kit (NZYTech). Amplification was performed in a Light Cycler 480 and data was analysed by using the comparative C_T_ method. Primers used for Hakai amplification were F-CGCAGACGAATTCCTATAAAGC and R-CCTTCTTCATCACCAGGTGG and as control to monitor loading difference, RPL13A levels were measures using the following primers F-CAAGCGGATGAACACCAAC and R-TGTGGGGCAGCATACCTC.

### Tumour xenograft in nude mice

Groups of athymic 6 weeks old mice (BALB/c, *nu/nu*) were used for xenograft assay. Mice were in a 12/12 hours light/dark cycle with water and food available ad libitum. Animals were always randomly distributed among the experimental groups. Five million MDCK or MDCK-Hakai cells 100 µl DMEM without serum and antibiotic were injected subcutaneously into the flank (*n* = 4 mice for MDCK cells and *n = *5 mice for Hakai-MDCK cells). Tumour volume was measured twice a week and animals were sacrificed 38 days after inoculation. Tumour volume was calculated as pLW2/6^49^. Tumours, lungs, kidneys and livers were collected and then fixed in 4% PFA and embedded in paraffin blocks for histology and/or immunohistochemistry (IHC) analyses.

### Histology and Immunohistochemistry

Sections (4 µm) of tumours, lungs, kidneys and livers were deparaffinised, rehydrated and stained with haematoxylin and eosin (H&E) using standard procedures. Deparaffinised and hydrated sections (4 µm) of tumours were also used for immunohistochemistry. Antigen retrieval was carried out with citrate or EDTA buffer following datasheet instructions and endogenous peroxidase activity was inhibited with peroxidase blocking (DakoCytomation). Samples were blocked for 1 hour at room temperature with 0,2%BSA and 0,1% Tx-100 and incubated overnight at 4 °C in a wet chamber with the primary antibody. Then, slides were incubated for 1 hour at room temperature with the secondary antibody and were revealed with DAB (DakoReal Envision kit). Then, slides were counterstained with Gill’s Hematoxylin and mounted with DePeX (Serva). Pictures were taken with an Olympus microscope in the indicated objectives in figure legends. Sections of tumours and teratoma stained with H&E were used for number of mitosis quantification. The number of mitosis was counted in 10 high magnification fields (objective 40x) of each tumour or teratoma with an Olympus BX50 microscope as previously reported^50^. Results are represented as mean ± SEM and a representative photograph was taken of each condition. Twenty formalin-fixed and paraffin-embedded (FFPE) colon cancer tissues (4 µm) of the I-IV TNM stages of colon cancer progression, ten adenomas and the corresponding adjacent normal colon tissues from the same patients were used for immunochemistry with anti-Hakai antibody. Five pictures of each section were taken with an Olympus microscope and the positive staining was quantified with Image J programme. Results are expressed as mean ± SEM of each TNM-stage of the disease and are represented in a scatter plot graphic.

### Quantification of lung invasion from *in vivo* mouse model

The presence of tumour cells in the lung mice was studied by real-time PCR^51^ using primers for HA epitope and Hakai present in ectopic HA-tagged Hakai expressed in MDCK-Hakai cells (5′-TCTGGGACGTCGTATGGGTA-3′; 5′-TTCTTCATCACCTTGCGGG-3′). Primers for mouse apolipoprotein B (*apob*) (5′-CGTGGGCTCCAGCATTCTA-3′; 5′-TCACCAGTCATTTCTGCCTTTG-3′) were used as endogenous control^51^. MDCK and Hakai-MDCK cell lines were used as negative and positive controls, respectively. Lung DNAs were obtained with QIAamp DNA Mini Kit (Qiagen) as previously described^52^ and the quality and quantity of extracted DNA was determined by using Nanodrop ND-spectrophotometer (Thermo Fisher Scientific, MA, USA). The amplification and quantification of 100 ng DNA was carried out in technical triplicates by using a LightCycler 480 real-time lightcycler (Roche). Data analysis was performed and relative levels of expression were calculated by 2^−ΔΔCt^ method^53^.

### Statistical analysis

Statistical significance of data was determined by applying a two-tailed Student t-test. Shapiro-Wilk test was used to check a normal distribution and Levene test to assess the equality of variances. Results obtained are expressed as mean ± SD or mean ± SEM. Quantification of human IHQ did not follow a normal distribution therefore we used Kruskal-Wallis with Tukey correction test. Significance of the Student t-test and Kruskal-Wallis with Tukey correction test among the experimental groups indicated in the figures is shown as * P < 0.05, **P < 0.01 and ***P < 0.001. Survival graphic in xenograft assay was made with GraphPad Prism software and the test of Breslow was used to calculate the *p* value.

## Electronic supplementary material

Supplementary Information
