# Supplementary material for: Hakai overexpression effectively induces tumour progression and metastasis in vivo
Source: Sci Rep. 2018 Feb 22;8:3466. doi: 10.1038/s41598-018-21808-w (PMC5823865; doi:10.1038/s41598-018-21808-w)
Supplement: Supplementary file 1 — Supplementary Information [file 41598_2018_21808_MOESM1_ESM.pdf]

Hakai overexpression effectively induces tumour progression and metastasis *in vivo*

Raquel Castosa[1†], Olaia Martinez-Iglesias[1†], Daniel Roca-Lema[1†], Alba Casas-Pais[1], Andrea Díaz-Díaz[1], Pilar Iglesias[1,2], Isabel Santamarina[3], Begoña Graña[3], Lourdes Calvo[3], Manuel Valladares-Ayerbes[4], Ángel Concha[2], and Angélica Figueroa[1*]

Supplementary Figures Legend

Supplementary Figure 1: Immunostaining of specific markers in human colon tissues. (A) Representative immunoreactivity of N-Cadherin, E-Cadherin and Cortactin in human samples from normal colonic mucosa, adenoma, and colorectal cancer (TNM stages I-IV). Images were obtained with a 20x objective. **(B)** Statistical quantification of N-Cadherin, E-Cadherin and Cortactin staining intensity in epithelial cancer cells at different colon cancer stages and in adenoma and normal colon tissues (normal colonic mucosa, n = 8; adenoma, n = 2; colorectal cancer, n = 8 of all stages). Five photographs of each tissue were quantified. Data are represented as bar plot. Values are means ± SD of staining intensity signal scoring per area. Calibration and quantification of the images were performed with ImageJ software. Kruskal-Wallis with Tukey correction test analyses show statistical differences in colorectal cancer (TNM, SI-IV) respect to paired healthy samples (*p <0.05; **p < 0,01; ***p <0,001). Scale bar 50 µm.

Supplementary Figure 2: Hakai induces EMT, oncogenic potential and invasion in normal epithelial cells. (A) MDCK epithelial cells and two representative clones of stable transfection of Hakai in MDCK cells (clone 4 and clone 11). Contrast phase images were obtained with a Nikon Eclipse-TI microscope using 20X objective. **(B)** Western blot analysis for E-cadherin, N-cadherin, Hakai and Cortactin in the indicated clones of Hakai-MDCK cells and normal MDCK cells. GAPDH and Tubulin are shown as loading controls. **(C)** Cell invasion chamber was performed to study invasion in MDCK cells and Hakai-MDCK cells, as described in Materials and Methods. Representative images were taken with an Olympus microscope using the 20X objective (*upper panel*) and quantification of the photographed invasive cells are represented as average mean ± SD of three independent experiments (*bottom panel*). **(D)** Hakai-MDCK cells (clone 4) were grown as colonies in soft agar, whereas MDCK cells do not form any colony.

After 21 days, colonies were counted as indicated in Materials and Methods. Representative photographed wells are shown (*Upper panel*) and quantification of the experiment was performed in triplicates and represented as mean ± SD of three experiments (*Bottom panel*) (**P < 0,01; ***P < 0,001).

Supplementary Figure 3: Full-length blots from Supplementary Figure 1. (A) Full-length blots analysis for E-cadherin, Hakai, Cortactin is shown in the indicated clones of Hakai-MDCK cells and normal MDCK cells. **(B)** Full-length blots analysis for N-cadherin in the indicated clones of Hakai-MDCK cells and normal MDCK cells. GAPDH and Tubulin are shown as a loading control.

Supplementary Figure 4: Immunohistochemistry with anti-HA antibodies. Representative images of immunohistochemistry with HA antibody (12Ca5, Roche) (left panel) or HA antibody (26183, ThermoFisher Scientific) (right panel) in tumours originated by Hakai-MDCK cells. Non-specific immunoreactivity was observed. Both tissues, connective tissue and tumours originated by Hakai overexpressing in Madin-Darby Canine Kidney (MDCK) cells, were stained by using these HA-antibodies. Connective tissue is in the right of the image, separated by the dashed line and marked with arrowheads, and tumour cells are in the left of the images, separated by the dashed line. Images were obtained with a 10x objective. Scale bar 100 µm.

Supplementary Figure 5: Low magnification images of immunohistochemistry from figure 4. **(A)** Representative images of immunohistochemical staining for Hakai and E-cadherin in teratomas or tumours originated by MDCK and Hakai-MDCK cells. **(B)** Representative immunoreactivity of N-cadherin and Cortactin in teratomas or tumours originated by MDCK or Hakai-MDCK cells. Images were taken with a 10x objective. Scale bar, 100 µm.

Supplementary Figure 6: Immunohistochemistry with anti HA antibody in tumour, lung and liver from nude mice. Representative images of immunohistochemistry with HA antibody in lungs and liver of mice inoculated with MDCK (left panel) and Hakai-MDCK (right panel) cells. Non-specific immunoreactivity in blood cells is observed. Images were obtained with a 20x objective. Scale bar 125 µm.

MATERIAL AND METHODS

Antibodies and materials

The rabbit polyclonal anti-Hakai antibody (Hakai-2498) was provided by Dr. Fujita. Antibody to cytoplasmatic domain of E-cadherin was from BD Transduction laboratories (610182) and was used for western blot. Anti-GAPDH (39-8600) was from Invitrogen and anti-α-Tubulin was from Sigma-Aldrich (St. Louis, MO). Anti-Cortactin antibody (05-180) was from Millipore. Anti-N-cadherin (ab18203) was from Abcam. HRP-rabbit (NA934) and mouse (NA931) polyclonal antibodies were from GE Healthcare. For western blot, primary antibodies were used at dilution 1:1000 and secondary antibodies at 1:5000. For human immunohistochemistry, 1/100 dilution was used for E-cadherin and Cortactin antibodies and 1/50 for N-cadherin.

Cell lines

MDCK cells were cultured in Dulbecco´s Modified Eagles Medium (DMEM) containing 1% penicillin/streptomycin, 1% glutamax and 10% of heat-inactivated fetal bovine serum (FBS). MDCK stably expressing Hakai cells (Hakai-MDCK clone 4 and clone 11) were provided by Dr. Fujita[17] and were cultured in presence of the selection antibiotic G418 (800 mg/ml). Cells were growth at 37°C in a humidified incubator with 5% CO_2. Cells were also tested regularly for mycoplasma contamination and all cells used were negative for mycoplasma test. For phase-contrast images, culture cells were photographed with a Nikon Eclipse-TI microscope.

Histology and Immunohistochemistry

Deparaffinised and hydrated sections (4 µm) of tumour, lung and liver from mice were used for immunohistochemistry with two different anti-HA antibodies (12CA5 from Roche and 26183 from Thermo Fosher). Anti-HA antibodies were used at 1/100 dilution. For human colon cancer tissues, deparaffinised and hydrated sections (4 µm) were used for immunohistochemistry with Anti-E-cadherin antibody (24E10), used at dilution 1/400, Anti-N-

cadherin antibody (ab18203), dilution 1/50, and with anti-Cortactin antibody (05-180), dilution 1/100. Antigen retrieval was carried out with citrate buffer in a pressure pot and immunohistochemistry was performed as indicated in material and methods of the manuscript. Pictures were taken with an Olympus microscope with the 20x objective.

Western blotting

For western blotting, $3x10^5$ MDCK or Hakai-MDCK cells were plated in 60mm dishes and after 24h the whole cell extracts were prepared as described previously[17]. Briefly, cells were lysed for 30 min in 1% Triton X-100 lysis buffer (20 mM Tris-HCL [pH 7.5], 150 mM NaCl, and 1% Triton X- 100) containing 2 mM leupeptin, 50 mM phenylmethylsulfonyl fluoride, and 80 µM aprotinin. After centrifugation at 12.000 rpm for 15 min, 20 µg of the supernatants were loaded in 10% polyacrilamide SDS-PAGE. Western blotting was performed as previously described[17].

Soft agar-colony formation assay

Soft agar-colony formation assay was performed on 12-well plates in triplicates at a density of $5x10^3$ MDCK or MDCK-Hakai cells/well in a medium containing 0,5% agarose layered over 0,6% agarose. Each well was allowed to solidify. After twenty-four hours, each well was covered with 150 µl culture media and was refreshed every 3 days. After 21 days cells were stained with 0,2% crystal violet. Colonies were counted manually in five fields of each well with a Nikon Eclipse TS100 microscope using a 4x objective and whole wells were photographied. Experiments were conducted in triplicates, repeated three times and are expressed as mean ± SD.

Invasion assay

For invasion assays, cells were previously cultured for 24 h with 1% FBS and 3x105 MDCK or

MDCK-Hakai cells were seeded in a cell invasion chamber (Cell invasion assaykit, Chemicon International) containing medium with 2% FBS plated in 24-well plate. Invasive cells migrate through a membrane for 16 hours, according to the gradient of FBS to the lower chamber that contains 30% FBS. Filters were fixed and stained with crystal violet following the manufacturer's specifications. Cells were counted by photographing five fields of the membrane through the Olympus microscope using a 20x objective. We performed triplicates of each condition and the assay was repeated at least three times. Results are expressed as mean ± SD.

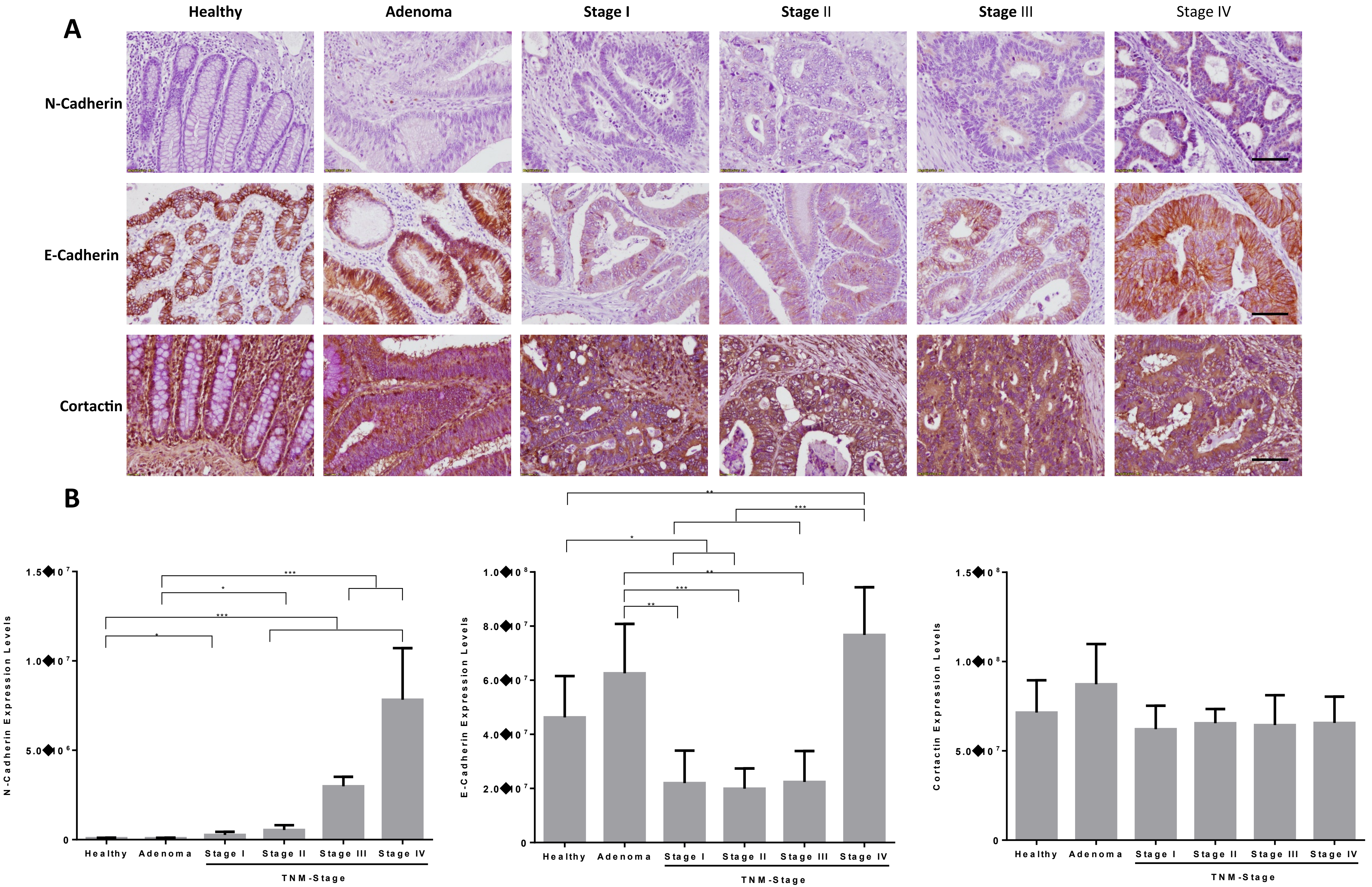

Supplementary Figure 1

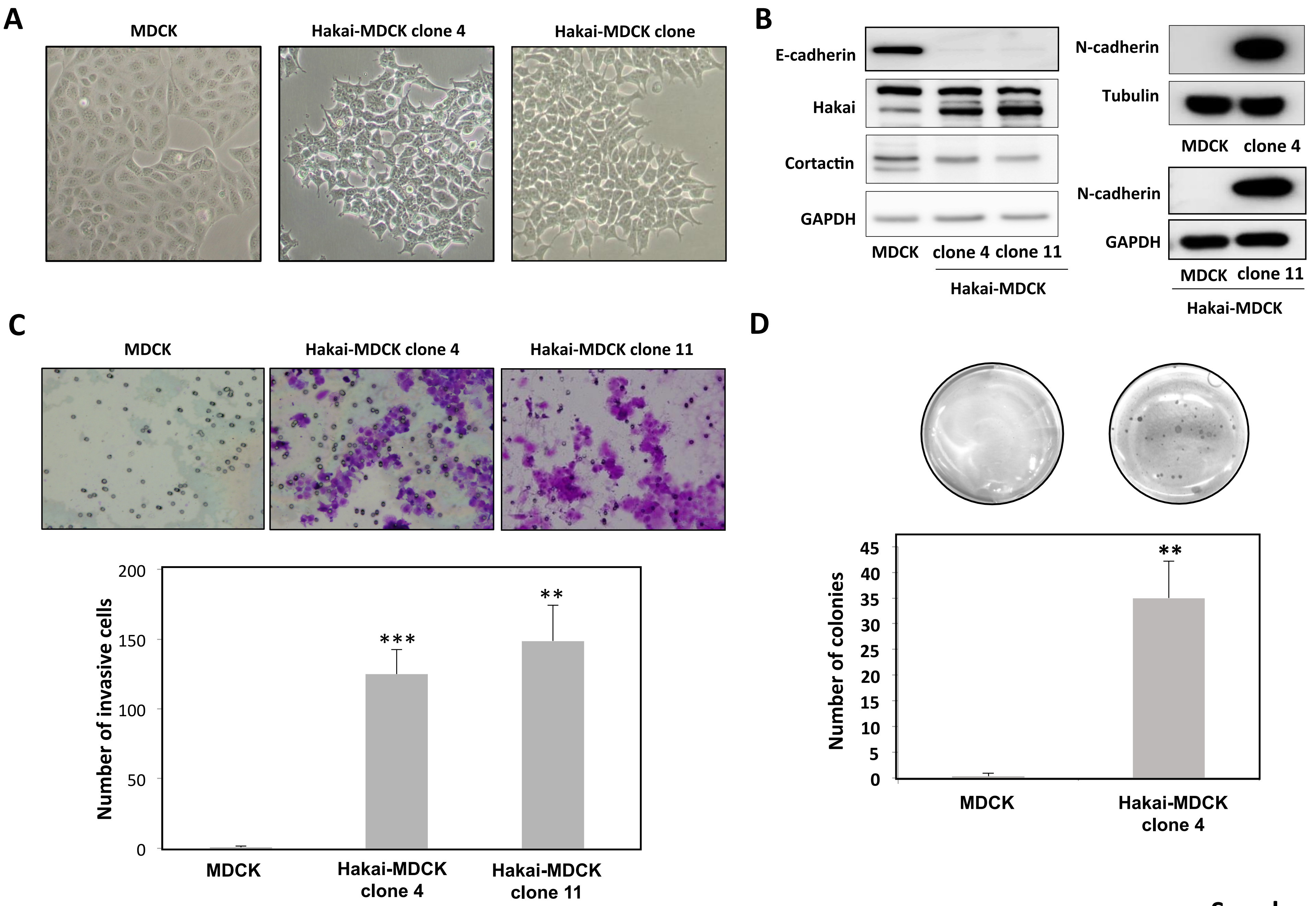

Supplementary Figure 2

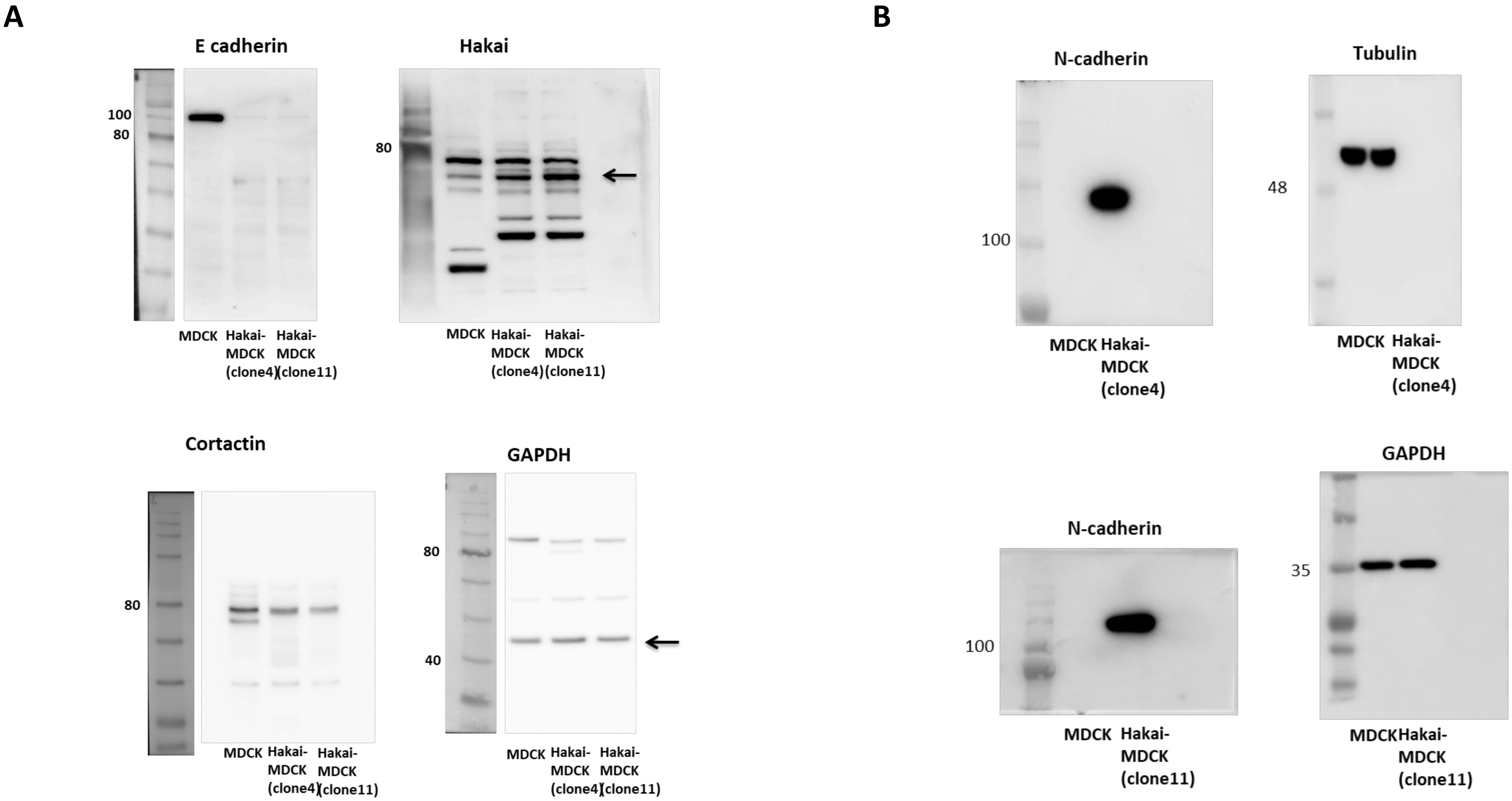

Supplementary Figure 3

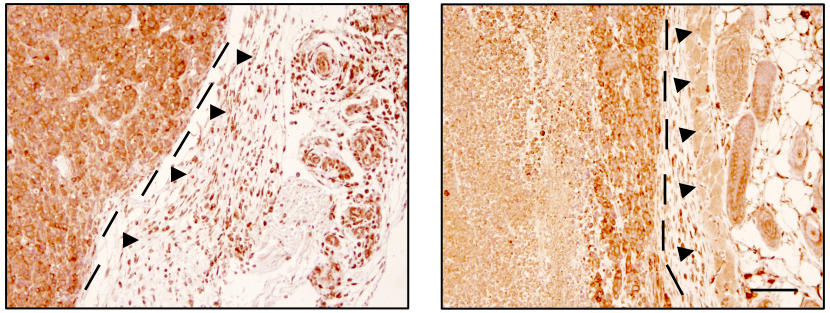

Anti-HA antibody
(Roche)

Anti-HA antibody
(Thermo Fisher)

Supplementary Figure 4

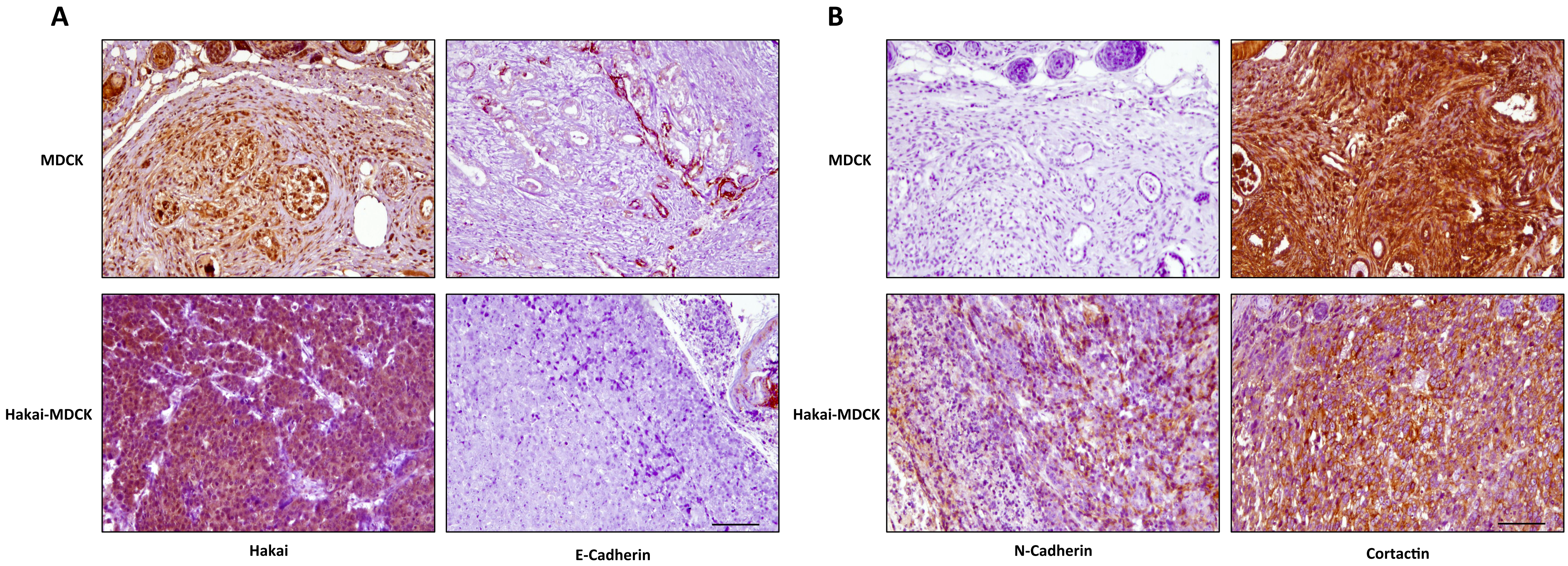

Supplementary Figure 5

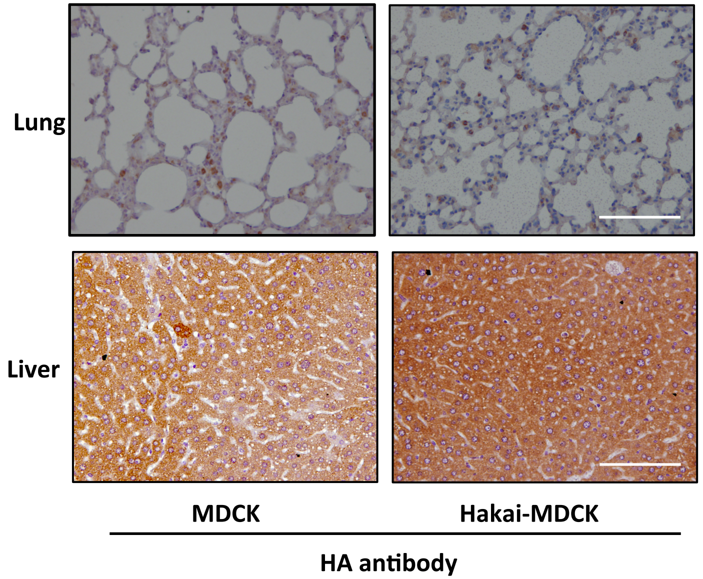

Supplementary Figure 6
